# DLCS-YOLO Model for Detecting Defects in Long-Distance Oil and Gas Pipelines

**DOI:** 10.3390/s26144523

**Published:** 2026-07-16

**Authors:** Yanan Wang, Rui Li, Kuan Fu, Tao Ma, Jie Huang, Jinyao Duan, Enpeng Wang, Ziyang Wang

**Affiliations:** 1 PipeChina Institute of Science and Technology, Tianjin 300457, China; 2 College of Mechanical and Transportation, China University of Petroleum, Beijing 102249, China

**Keywords:** permanent magnetic field perturbation, pipeline defect detection, small-target detection, YOLOv11, deep learning

## Abstract

This study developed the Deformable Large-kernel Context-fused Spatial (DLCS)-YOLO model to address various challenges involved in permanent magnetic field perturbation (PMFP)-based defect detection for long-distance oil and gas pipelines, including a high false-positive rate, susceptibility to background noise interference, difficulty in identifying small-scale defects, low precision in feature representation and defect type discrimination, and poor adaptability to multiscale defects. The proposed model is an improved version of You Only Look Once (YOLO) v11n. The backbone of the proposed model contains the C3k2-Deformable Attention (C3k2-DAttention) module and the Spatial Pyramid Pooling-Fast-Large Separable Kernel Attention (SPPF-LSKA) module, which is used in place of the SPPF module to enhance robustness to noise and fine-grained feature extraction for small-scale defects. In the feature fusion layer, the Context-Guided Feature Pyramid Network (Context-Guided FPN) module is used to replace the conventional concatenation operation, thereby improving feature representation and defect classification accuracy. Furthermore, the Spatially Enhanced Attention Module (SEAM) is incorporated into the detection head to enhance adaptability in complex scenarios, including those involving background interference and multiscale defects. Experimental results indicate that the proposed model achieves mAP@50 and mAP@50:95 values of 94.5% and 64.7%, respectively, on a self-constructed dataset, with a computational cost of only 6.2 GFLOPs. Compared with the baseline YOLOv11n model, the proposed model exhibits a 3.1% higher precision, a 3.8% higher mAP@50 value, and a 3.0% higher mAP@50:95 value and requires 0.1 fewer GFLOPs. The proposed algorithm effectively enhances the accuracy and efficiency of pipeline defect detection, demonstrating considerable practical value and broad application prospects for detecting defects in oil and gas pipelines.

## 1. Introduction

Oil and gas pipelines are core components of national energy transmission systems. Therefore, the operational reliability of these pipelines directly affects national energy supply stability and is closely related to ecological and environmental protection as well as public safety [1]. With the aging of long-distance oil and gas pipelines, the complex evolution of geological environments along these pipelines, and the increasingly severe corrosion caused by the transported media, hidden hazards caused by pipeline defects, including microcracks and metal loss, have become increasingly prominent. This has thus increased the likelihood of major safety incidents [2]. PMFP signals are still primarily analyzed and interpreted manually; manual methods are highly susceptible to subjective factors and prone to problems such as false detections and missed detections of defects [3]. Accordingly, the development of deep-learning-based object detection algorithms for the high-precision identification of pipeline defects has become essential for addressing the aforementioned problems [4].

Deep learning has attracted considerable research attention because of its advantages in automatic feature extraction from data. Numerous studies in the field of pipeline defect detection have comprehensively explored algorithm optimization to address challenging detection scenarios, including those involving complex background interference, masked defect features, small-scale defects, and image noise. For example, Yao et al. [5] proposed the TWSC intelligent diagnosis model based on transfer learning and feature fusion. This model integrates the multifeature distributions of the continuous wavelet transform and short-time Fourier transform to enhance anti-interference robustness for oil and gas pipelines under complex working conditions and sample perturbations, and it achieved an accuracy of 96.0% on a validation set. Xu et al. [6] proposed an algorithm based on YOLOv7 for detecting defects in pipeline welds. In this algorithm, the Coordinate Attention mechanism is embedded into the backbone network to integrate positional information and channel attention, thus effectively suppressing interference from irrelevant features, such as the pipeline background and weld texture. The aforementioned algorithm achieved an mAP@0.5 value of 78.6%, which was 15.9% higher than that of the baseline YOLOv7 algorithm. Yang et al. [7] proposed the OFG-YOLO algorithm, which incorporates improvements in feature extraction optimization, multiscale fusion, and sample generalization. Specifically, the original SPPF module in YOLOv8 was replaced with a Focus Modulation Module, to amplify core defect features and suppress irrelevant background interference. The OFG-YOLO model achieved an mAP@0.5 of 80.7%. Chen et al. [8] proposed a hybrid detection model that combines YOLOv5 and the Vision Transformer (ViT). This model leverages the global attention of the ViT to capture the global features of defects, thereby avoiding interference from local noise and signal fluctuations and achieving a favorable trade-off between detection efficiency and accuracy (overall accuracy of 96.75%). In the aforementioned studies, irrelevant features were suppressed, and noise was filtered out through strategies such as feature fusion, attention mechanism embedding, and hybrid architecture design, thereby considerably improving detection stability and reliability in complex scenarios. Pipeline defects typically exhibit multiscale characteristics and show weak feature representation at small scales; thus, small pipeline defects are easily missed, which limits detection accuracy. Zhao et al. [9] introduced an inverted residual mobile module into the backbone network of YOLOv8 to enhance its feature representation ability, and they replaced the convolutional layers in the neck of this model with GSConv layers to conduct multiscale feature fusion; the improved model achieved an mAP of 95%. Chen et al. [10] constructed a pipeline defect detection system by combining a cycle-consistent generative adversarial network (CycleGAN) model and an improved YOLOv5 model. First, CycleGAN was used to augment the defect dataset and balance defect categories to mitigate model overfitting. The Transformer attention mechanism was then incorporated into the YOLOv5 model to enhance the extraction of small-scale defect features in complex backgrounds. These improvements led to the aforementioned detection system having a 2.53% higher accuracy than the original YOLOv5 model. Shen et al. [11] proposed the EFE-SSD algorithm, which uses a dense skip connection module to prevent the loss of small-target features as the network deepens, thereby improving the utilization and discriminability of these features and achieving a 2.26% higher average precision than the SSD model. Xiong et al. [12] proposed a Hybrid CNN-Mamba Network (HCMNet), which combines the local-detail extraction capability of convolutional neural networks (CNNs) with the global contextual modeling capability of Mamba. This network uses a wavelet feature extraction module and an adaptive feature fusion module to enhance object segmentation performance under complex backgrounds. Xiong et al. [13] proposed a Cascade Fusion Network for road extraction (CFRNet), which strengthens multiscale feature interaction through a multilevel cascade structure and a three-layer adaptive feature fusion module, thereby improving the extraction of slender targets and occluded regions in remote sensing road extraction tasks. The aforementioned algorithmic improvements effectively enhanced the detection accuracy of multiscale defects, providing diverse technical approaches and practical references for pipeline defect detection.

YOLOv11 is a high-performance object detection algorithm that exhibits strong backbone feature extraction performance, high multiscale feature fusion efficiency, and robust small-target detection performance. Its lightweight network architecture and efficient feature modeling capability make it an ideal baseline model for PMFP-based detection of defects in long-distance oil and gas pipelines. This study developed a DLCS-YOLO model, in which YOLOv11n serves as the baseline model. The baseline model is improved by optimizing feature extraction in its backbone network, enhancing its multiscale feature fusion architecture, and strengthening the performance of its detection head. The proposed model was designed to address various challenges in PMFP-based defect detection in long-distance oil and gas pipelines, including high false-positive rates, strong background interference, difficulty in identifying small-scale defects, low precision in feature representation and defect type discrimination, and poor adaptability to multiscale defects.

## 2. Materials and Methods

### 2.1. Principle of PMFP-Based Defect Detection

**Figure 1:**
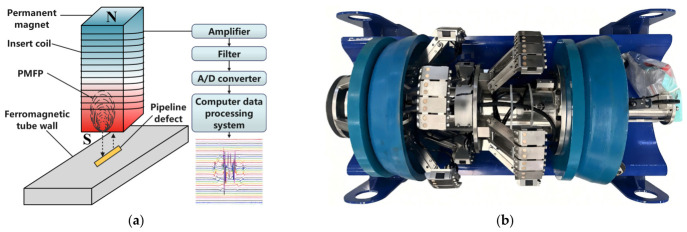
Principle and photograph of an ILI device used to detect PMFP signals for pipelines. (**a**) Schematic of the detection of permanent magnetic field perturbation (PMFP) signals for pipelines; (**b**) Image of a specialized ILI tool used for pipelines(PipeChina Institute of Science and Technology, Tianjin, China). In (**a**), the blue and red regions of the permanent magnet indicate the two opposite magnetic poles, the gray region represents the ferromagnetic tube wall, and the yellow rectangle denotes a pipeline defect. The dashed arrows indicate the direction of the PMFP signal.

Nondestructive testing (NDT) of long-distance oil and gas pipelines is the core component of pipeline integrity management, and an in-line inspection (ILI) device is the core equipment for pipeline defect detection. ILI technologies for pipelines are classified into three major categories: single-principle detection, composite integrated detection, and emerging detection technologies. The use of detection technologies based on magnetic flux leakage and eddy current has become widespread in industry because they do not require a coupling agent and exhibit strong engineering adaptability [14]. To enhance the detection performance of inspection tools, multiprinciple fusion-based coupled inspection technologies have been developed, with their accuracy improved through methods such as magnetoelectric integration [15]. In the present study, PMFP was adopted as an efficient NDT technique for steel oil and gas pipelines. This technique enables accurate identification of typical pipeline defects, including metal loss, dents, and patches. The technique is based on the magnetic coupling effect between permanent magnets and ferromagnetic pipelines as well as the mechanism of magnetic field distortion at defects. It requires no additional excitation power supply and offers substantial advantages in terms of device compactness and energy efficiency. The detection probe consists of a permanent magnet that serves as the magnetic excitation source, with an enameled wire induction coil wound around its surface (Figure 1) [16]. When the probe moves at a constant speed along the inner wall of a pipeline, a stable magnetic field is established between the probe and the pipe wall. In defect-free areas, the coil’s output signal remains approximately constant. However, when the probe passes through different types of defects or pipeline components, differentiated magnetic field responses are generated. The acquired perturbation signals are amplified, filtered, and then subjected to analog-to-digital conversion before being transmitted to a computer. Subsequently, accurate identification and localization of defects are achieved on the basis of the relationship between defect type and signal characteristics. The aforementioned method enables the assessment of the integrity of oil and gas pipelines and is particularly suitable for rapid pipeline inspection and periodic monitoring. The special-purpose ILI tool used to examine pipelines (Figure 1) is mainly composed of a bumper plate, odometer wheels, a junction box, a PMFP probe, a sealing cup, a support plate, and a front bumper [17].

### 2.2. Improvement of YOLOv11

**Figure 2:**
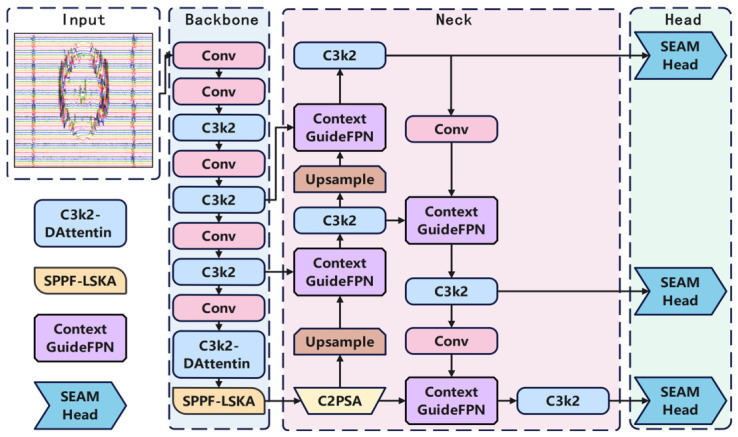
Schematic of the proposed DLCS-YOLO model. Different colors are used to distinguish different modules. Solid arrows indicate the direction of feature propagation, while dashed outlines divide the network into three main stages: the backbone, neck, and detection head. The four modules shown separately on the left are the improved modules in this work.

YOLOv11 inherits the single-stage detection advantages of the YOLO series, and its optimized backbone network provides stronger potential for multiscale feature extraction while maintaining a very low parameter count. This characteristic is crucial for practical engineering applications in which ILI devices for long-distance oil and gas pipelines are often constrained by edge-computing hardware. To effectively address the challenges associated with PMFP-based defect detection for long-distance oil and gas pipelines, including low detection accuracy, a high false-positive rate, susceptibility to background noise interference, difficulty in identifying small-scale defects, low precision in feature representation and defect type discrimination, and poor adaptability to multiscale defects, YOLOv11n was selected as the baseline model in this study, and the following improvements were made (Figure 2). First, the C3k2-DAttention module is introduced into the backbone network of YOLOv11, and the original SPPF module is replaced with the SPPF-LSKA module. These modifications enhance the modeling capability of key regional features, expand the receptive field, and improve the model’s ability to capture small-target features while suppressing complex background interference. Second, the Context-Guided FPN module is used to replace the conventional concatenation operation in the neck structure, thereby optimizing the semantic guidance and fusion of multiscale defect features and improving the complementarity and consistency of cross-scale defect representations. Finally, the detection head is redesigned using the SEAM. By leveraging the synergistic effect of multiscale receptive fields, this module accentuates defect-related channel features and suppresses background interference, thereby effectively reducing computational redundancy while substantially improving the detection accuracy for small defects.

### 2.3. C3k2-DAttention Module

**Figure 3:**
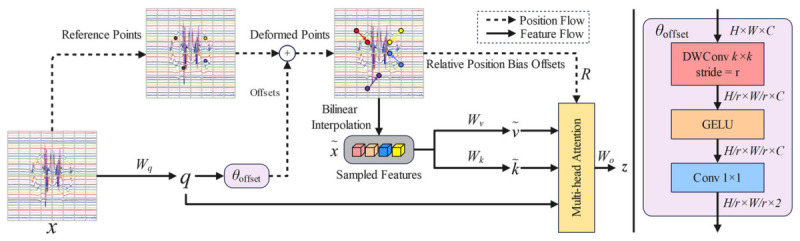
Deformable Attention module. Solid arrows indicate the Feature Flow, while dashed arrows indicate the Position Flow.

Defects of oil and gas pipelines have irregular morphologies, and the PMFP signals of these defects are susceptible to noise and background interference. Traditional convolutional neural network models exhibit insufficient detection performance for irregular defects and weak anti-interference capability [18]. By contrast, Transformer-based models exhibit strong performance in these areas [19]. To enhance the detection of irregular defects and anti-interference capability, this study introduced DAttention [20] into selected C3k2 modules in the backbone of the YOLOv11 model. DAttention (Figure 3) is an efficient deformable self-attention mechanism that can dynamically optimize a model’s attention weight allocation for key regions on the basis of the feature distribution of the input data. In contrast to the attention mechanism in traditional Transformers, which applies dense global attention across all positional information, DAttention focuses more on the target defect regions within an image, which effectively reduces interference from irrelevant backgrounds and improves the model’s anti-interference capability.

### 2.4. SPPF-LSKA Module

**Figure 4:**
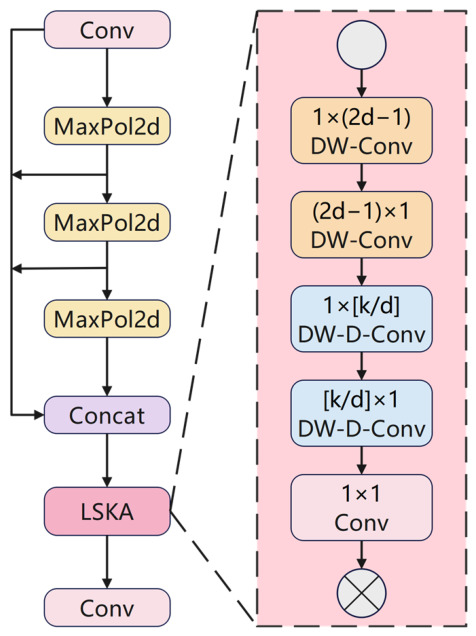
Architecture of the SPPF-LSKA. Solid arrows indicate the direction of feature flow, while dashed lines indicate the internal structure of the LSKA module.

SPPF is an improved version of the Spatial Pyramid Pooling module that adaptively fuses features from different receptive fields through multiscale pooling, thereby considerably enhancing the adaptability and robustness of deep learning models to object scale variations. However, the SPPF module exhibits limitations in capturing fine-grained features. The continuous pooling process leads to the loss of pixel-level detailed information, which is particularly crucial in small-object detection scenarios. Large Separable Kernel Attention (LSKA) [21] is an improved version of Large Kernel Attention [22] that combines the advantages of a large-kernel receptive field and separable convolution. Although traditional large-kernel convolutions can capture global information and large-scale features, they have limited capability in representing small details, which reduces the overall detection performance of the model. To meet the demand for detecting small defects in oil and gas pipelines, this study replaced the original SPPF module in YOLOv11 with the SPPF-LSKA module that incorporates LSKA into the SPPF block (Figure 4). This replacement enhances the feature response in key regions, improves detection accuracy for small targets, and reduces the probability of false positives.

### 2.5. Context-Guided FPN Module

**Figure 5:**
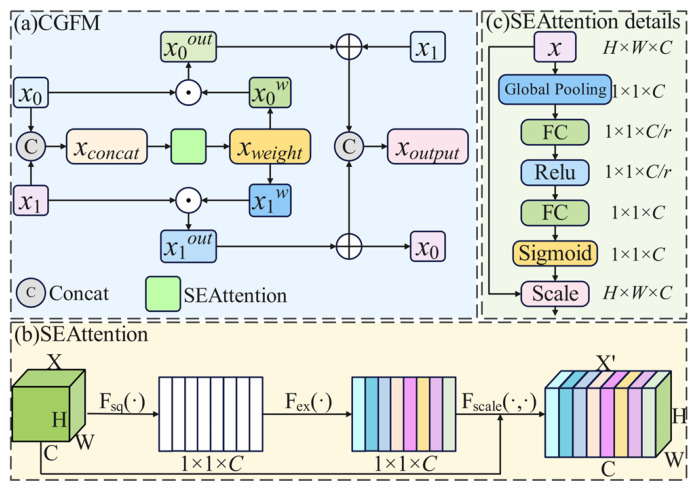
Architecture of the CGFM. Solid arrows indicate the direction of feature flow, while the dashed outlines in (**b**,**c**) show the detailed submodules of CGFM.

Although the concatenation feature fusion method adopted by YOLOv11 can efficiently integrate multiscale features, this method only directly concatenates feature maps in the channel dimension without assigning weights to features of different levels. This approach results in the masking of fine-grained feature details, information redundancy, and fusion imbalance, ultimately restricting detection accuracy. To address the aforementioned problems, this study replaced the traditional concatenation feature fusion mechanism in YOLOv11 with the Context-Guided FPN module. This module not only improves semantic consistency among multiscale features but also effectively enhances the model’s ability to preserve fine-grained details. The Context-Guided FPN module consists of three components, namely those conducting backbone feature extraction, pyramid path propagation, and context-guided feature fusion. Its core functional submodule is a context-guided fusion submodule (CGFM) (Figure 5), which addresses the problems of insufficient cross-channel interaction and substantial semantic discrepancies in traditional fusion methods. The Context-Guided FPN module effectively improves feature representation capability and the precision of defect type discrimination, thus representing a key improvement over the concatenation layer.

### 2.6. SEAM

**Figure 6:**
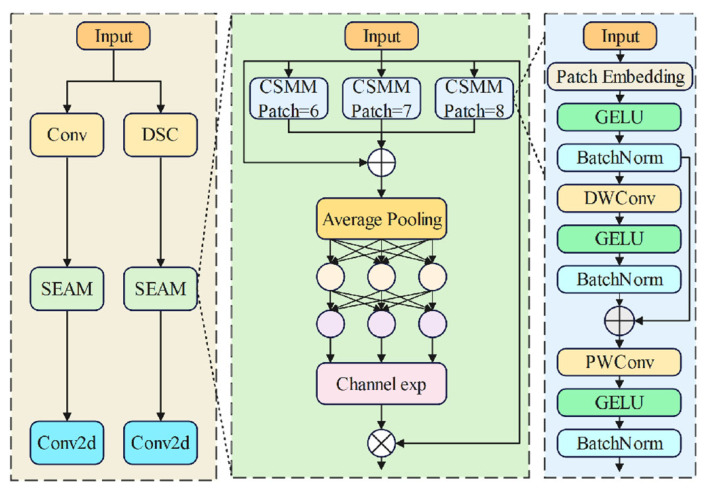
Architecture of the modified head of YOLOv11 (with the SEAM). Solid arrows indicate the direction of feature propagation, while the dashed lines on the right lead out the internal structure of the SEAM module.

To address the feature ambiguity caused by multiscale feature processing and background interference in complex scenarios, this study introduced the SEAM into the detection head of YOLOv11 (Figure 6) [23].

The attention weights generated by the SEAM are fused with the original feature maps through element-wise weighting, which enhances the responses of defect-related channels and suppresses background interference. The multiscale receptive fields embedded in the SEAM operate synergistically to adapt to defects of different sizes. Large receptive fields capture the global trend and reduce misjudgments caused by noise, whereas small receptive fields capture detailed features, thus increasing the detection probability of small defects. The SEAM is placed after the depthwise separable convolutional layers of the detection head. This module maintains computational efficiency through its lightweight design and achieves refined feature enhancement, thereby improving the detection accuracy of pipeline defects under complex backgrounds in PMFP-based inspection.

## 3. Results

### 3.1. Experimental Environment and Parameters

The proposed DLCS-YOLO model was used to conduct pipeline defect detection experiments on an Intel Core Ultra 7 255HX 2.40-GHz central processing unit with a Windows 11 operating system and an NVIDIA GeForce RTX 5060 8 GB graphics processing unit (GPU). The experimental environment was configured using Python version 3.11.14 and PyTorch version 2.7.0, with CUDA 12.8 employed for GPU acceleration. The fixed experimental parameters are listed in Table 1.

To ensure the fairness of the comparison experiments, all models were trained and tested using the same dataset partition, input size, evaluation metrics, and hardware environment. Considering the differences in network architecture and training strategy between the YOLO-series models and RT-DETR-L/D-FINE-N, training parameters suitable for each model architecture were adopted, and all models were confirmed to reach stable convergence. For all models in the comparison experiments, three independent training runs were conducted with random seeds of 0, 42, and 65. The final detection results are reported as the mean ± standard deviation. During model evaluation, the official default post-processing thresholds were used, with a confidence threshold of 0.001 and an NMS IoU threshold of 0.7.

### 3.2. Dataset

**Figure 7:**
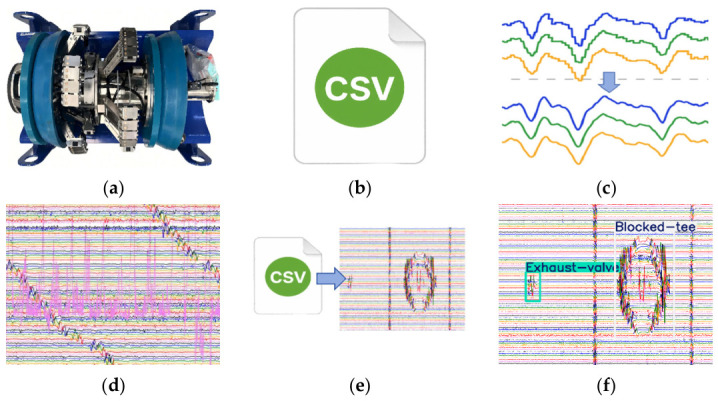
Workflow of PMFP data acquisition and inspection image generation. (**a**) PMFP data acquisition; (**b**) Data format conversion; (**c**) Mileage accuracy correction; (**d**) Abnormal signal filtering; (**e**) Inspection image generation; (**f**) Defect annotation.

The dataset used in this study was derived from actual PMFP inspection projects for in-service long-distance oil and gas pipelines. The workflow of PMFP data acquisition and inspection image generation is shown in Figure 7. First, the original PMFP inspection signals were collected using an in-line pipeline inspection device(PipeChina Institute of Science and Technology, Tianjin, China). The original data were then converted into a format in which mileage information, sensor numbers, and channel signal values could be used for subsequent processing. To improve the continuity and clarity of the signal curves in the inspection images, mileage accuracy correction was performed to reduce local stepped patterns and detail loss caused by mileage quantization over long cumulative distances. Abnormal signals caused by sensor anomalies or data transmission errors were then filtered to reduce their influence on inspection image quality and defect annotation. Finally, the processed PMFP signals were converted into inspection images, and the defect regions were annotated and reviewed by professionals [24].

To improve the consistency of defect annotation, labeling criteria for each defect category were established according to the PMFP signal morphology, channel response range, mileage position, and actual inspection records, as shown in Table 2. During annotation, background noise, random disturbances, or abnormal signals were not labeled as defect targets if they lacked stable local structural features and could not be matched with actual pipeline structure records. For samples with uncertain category judgments, the final category and bounding-box range were determined after joint review by the annotation engineer and the senior engineer.

**Figure 8:**
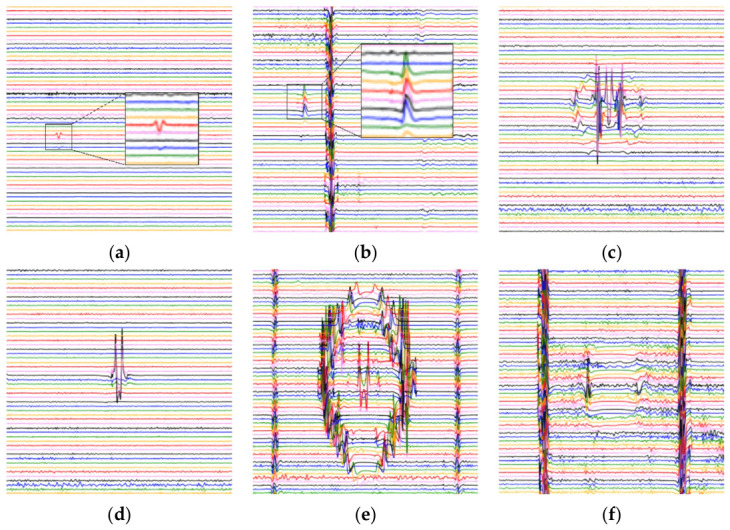
Representative samples from the adopted dataset. (**a**) Metal loss; (**b**) Indentation; (**c**) Patch; (**d**) Exhaust valve; (**e**) Blocked-tee; (**f**) Tee. The boxes in (**a**,**b**) show local enlarged regions.

The dataset contains 348 original images across seven categories: metal loss, indentations, patches, exhaust valves, blocked-tees, valves, and tees. The original images were randomly divided into a training set, validation set, and test set at a ratio of 7:2:1, with the distribution of each category maintained as reasonably as possible across the three subsets. To mitigate overfitting and avoid data leakage, offline data augmentation was performed independently after dataset partitioning, resulting in 753 images in the training set, 197 images in the validation set, and 102 images in the test set. All images were independently annotated using LabelImg by engineers with more than three years of pipeline inspection experience, and the annotations were subsequently reviewed by a second senior engineer to ensure consistency and accuracy. Some representative samples from the dataset are shown in Figure 8.

The number of images in each category and their distribution among the training, validation, and test sets are presented in Table 3. Data augmentation was performed using an AutoAugment strategy implemented with the Albumentations library, mainly consisting of affine transformations and color perturbations. The affine transformations included random rotation, scaling, and translation, with a rotation range of −20° to 20°, a scaling ratio of 0.9 to 1.1, a translation ratio of −10% to 10%, and an execution probability of 100%. The color perturbations included adjustments to brightness, contrast, saturation, and hue, with variation amplitudes of 0.2 for brightness, contrast, and saturation and 0.05 for hue, and an execution probability of 80%. During augmentation, the target bounding boxes and class labels were updated synchronously to ensure consistency between the augmented images and the annotation information.

**Figure 9:**
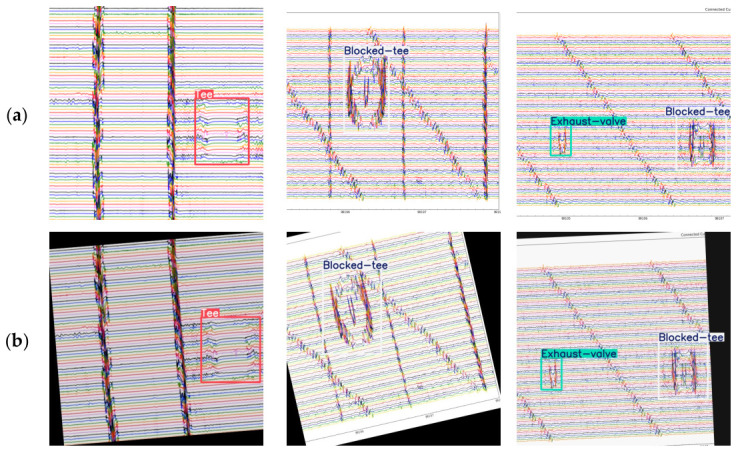
AutoAugment data augmentation examples. (**a**) Original image; (**b**) Image after AutoAugment augmentation.

The results of representative samples after applying the AutoAugment strategy are shown in Figure 9. The aforementioned image transformations do not alter the basic structure or category semantics of the defect regions. Instead, they simulate variations in imaging angle, sensor response intensity, and image contrast that may occur during actual inspection, thereby improving sample diversity. All augmented samples were manually checked to ensure the validity of defect morphology, class labels, and bounding-box locations.

### 3.3. Evaluation Metrics

This study selected precision (P), recall (R), F1-score, average precision (AP), mean average precision (mAP), parameter count (Params), and number of floating-point operations (FLOPs) as metrics for objectively evaluating the performance of the proposed model in pipeline defect detection. Precision represents the proportion of real defects among all samples classified as defects by a model, reflecting the accuracy of detection results. This parameter is calculated as follows:

(1) $$P=\frac{\mathrm{TP}}{\mathrm{TP}+\mathrm{FP}}$$

where TP and FP denote the numbers of correctly and incorrectly detected pipeline defects, respectively.

Recall represents the proportion of correctly detected defects among all actual defect samples, reflecting the model’s ability to detect defect targets. This parameter is calculated as follows:

(2) $$R=\frac{\mathrm{TP}}{\mathrm{TP}+\mathrm{FN}}$$

F1-score comprehensively considers precision and recall and is used to evaluate the balance between detection accuracy and defect detection capability. This parameter is calculated as follows:

(3) $$F1=\frac{2\times P\times R}{P+R}$$

Average precision (AP) represents the area under the precision-recall (P-R) curve for a specific category at different confidence thresholds and is used to evaluate the detection performance of a model for a single target category. This parameter is calculated as follows:

(4) $$\mathrm{AP}=\int_{0}^{1}P(R)\mathrm{dR}$$

mAP, which is a core performance metric in object detection tasks, represents the mean of average precision values calculated at different IoU thresholds. A higher mAP value indicates better detection performance. This parameter is calculated as follows:

(5) $$\mathrm{mAP}=\frac{\sum_{i=1}^{n}{\mathrm{AP}}_{i}}{n}$$

where *n* represents the number of categories in the dataset (*n* = 7), and APi is the average precision corresponding to the ith category. AP@50 represents the AP of a specific category at an IoU threshold of 0.50, whereas AP@50:95 represents the average AP calculated at IoU thresholds ranging from 0.50 to 0.95 with a step size of 0.05. mAP@50 and mAP@50:95 represent the mean AP@50 and mean AP@50:95 values across all categories, respectively. In this study, mAP@50 and mAP@50:95 were selected for performance evaluation.

The parameter count reflects the capacity and complexity of a model, with a larger parameter count indicating a more complex model. Moreover, the number of FLOPs represents the computational cost of a model, serving as a crucial indicator of its computational efficiency.

### 3.4. Ablation Experiment

To verify the effectiveness of each improvement made to the proposed model, ablation experiments were conducted in the configured experimental environment (Table 4). In these experiments, a comprehensive performance evaluation was performed using precision, mAP@50, mAP@50:95, parameter count, and number of FLOPs. In Table 4, the module combinations corresponding to each ablation configuration are directly listed to more clearly show the effects of different structural combinations on model performance. Comparative analysis indicated that all improved modules enhanced the model performance to varying degrees, with the optimal detection performance achieved when all modules were employed.

As presented in Table 4, with YOLOv11n used as the baseline model, the four improved modules produced distinct performance gains when introduced individually. When C3k2-DAttention was used alone, mAP@50 increased from 90.7% to 93.9% (+3.2%), and mAP@50:95 increased from 61.7% to 63.2% (+1.5%). Meanwhile, the parameter count increased only slightly from 2.583 million to 2.618 million, and the computational cost remained almost unchanged at 6.3 GFLOPs. This indicates that the DAttention module dynamically optimizes attention weight allocation, thereby significantly enhancing the model’s ability to focus on key defect regions and effectively suppressing background noise interference with almost no additional computational burden. These results verify its independent value in improving the quality of feature extraction. When SPPF-LSKA was used alone, mAP@50:95 increased to 62.2% (+0.5%), and the parameter count increased slightly to 2.856 million. Although the improvement in accuracy-related metrics was relatively moderate, this module integrates the large separable kernel attention mechanism, expands the receptive field, and strengthens the feature response in key regions. Therefore, it provides a feature basis for the subsequent performance improvement achieved by combining modules and demonstrates its independent role in enhancing small-target features. When Context-Guided FPN was used alone, mAP@50:95 increased to 62.5% (+0.8%). By replacing the conventional concatenation operation with a semantic guidance mechanism, this module effectively alleviates semantic discrepancies in cross-scale feature fusion and enhances the preservation of fine-grained features, verifying its independent contribution to improving feature fusion efficiency. When SEAM Head was used alone, the model exhibited remarkable lightweight characteristics. The parameter count decreased from 2.583 million to 2.491 million, and the computational cost decreased substantially from 6.3 to 5.8 GFLOPs. Meanwhile, mAP@50:95 increased to 62.2% (+0.5%), and precision also improved. Only mAP@50 showed a slight decrease of 0.4%, from 90.7% to 90.3%. This result can mainly be attributed to the fact that SEAM tends to improve bounding box regression accuracy under high IoU thresholds and filters out many prediction boxes that only satisfy the IoU = 0.5 criterion but exhibit coarse localization. Therefore, a slight decrease occurred under the relatively loose evaluation threshold, whereas better performance was achieved for the stricter mAP@50:95 metric, fully demonstrating its independent advantage in reducing false detections and improving localization accuracy.

After the independent contributions of each module were clarified, further combined experiments revealed the synergistic optimization mechanism among the modules. The addition of SPPF-LSKA to C3k2-DAttention further increased precision to 92.6%, with mAP@50:95 maintained at a high value of 63.1%. These two modules functioned in a complementary manner and stabilized the detection performance. The further introduction of Context-Guided FPN caused mAP@50 to increase to 93.7%. The semantic guidance of the Context-Guided FPN optimized the feature fusion efficiency and strengthened the complementarity of multiscale features. Notably, when SEAM Head was introduced on this basis, the first three modules effectively enhanced the model’s feature expression and object detection ability, thereby compensating for the influence of using SEAM Head alone on detection under the loose threshold of mAP@50. As a result, the model achieved the best performance across all metrics: mAP@50:95 increased substantially from 61.7% for the baseline model to 64.7% (+3.0%), mAP@50 increased to 94.5% (+3.8%), and P reached 93.0% (+3.1%). Meanwhile, the computational cost decreased to 6.2 GFLOPs, slightly lower than that of the baseline model, and the parameter count was controlled at 2.955 million.

In conclusion, the four modules formed a complete optimization chain involving enhanced feature extraction, receptive field expansion, semantic fusion optimization, and detection head refinement. Each module provided an independent performance gain while also producing positive synergistic effects. Ultimately, the proposed model achieved a substantial improvement in the stringent mAP@50:95 metric while maintaining a lightweight design and practical operational efficiency, making it well suited to the engineering requirements of PMFP-based defect detection for oil and gas pipelines.

### 3.5. Performance Comparison Experiments

#### 3.5.1. Comparison Between the Proposed Model and General-Purpose YOLO Models

To systematically evaluate the performance of the DLCS-YOLO model in PMFP-based pipeline defect detection, six general-purpose YOLO models, namely YOLOv5n, YOLOv8n [25], YOLOv10n, YOLOv11n, YOLOv12n, and YOLOv13n, were selected for comparison. Each comparison model was retrained and tested on the dataset used in this study based on its official implementation and recommended configuration.

In Table 5, P, R, F1, mAP@50, and mAP@50:95 are reported as the mean ± standard deviation of three independent training runs, and the small standard deviations of these metrics indicate that the results of repeated training were relatively stable. The proposed DLCS-YOLO model exhibited prominent performance in the core detection accuracy metrics for PMFP-based pipeline defect detection. In terms of mAP@50, the value achieved by DLCS-YOLO (94.5%) was 3.3%, 4.1%, 5.9%, and 3.8% higher than those of YOLOv5n, YOLOv8n, YOLOv10n, and YOLOv11n, respectively, and 8.0% and 6.8% higher than those of YOLOv12n and YOLOv13n, respectively. In terms of mAP@50:95, the value achieved by the proposed model (64.7%) was 4.3%, 3.0%, and 4.1% higher than those of YOLOv5n, YOLOv8n, and YOLOv10n, respectively, and 6.4% and 2.5% higher than those of YOLOv12n and YOLOv13n, respectively. Moreover, the P, R, and F1 values of the proposed model reached 93.0%, 90.1%, and 91.5%, respectively, further demonstrating the advantage of the proposed model in the detection accuracy of PMFP-based pipeline defects.

The proposed model required 6.2 GFLOPs, which was only slightly higher than the number of FLOPs required by YOLOv5n but lower than the number of FLOPs required by YOLOv8n and YOLOv10n. Although the parameter count of the proposed model (2.955 million) was moderately higher than those of some lightweight YOLO models, the proposed model exhibited the best trade-off between detection accuracy and computational efficiency.

**Figure 10:**
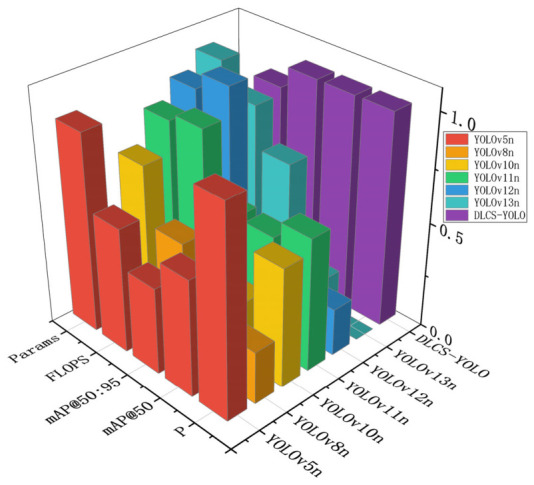
Three-dimensional column chart of the performance of the proposed DLCS-YOLO model and different general-purpose YOLO models in PMFP-based pipeline defect detection.

Figure 10 illustrates a three-dimensional column chart of the performance of the proposed DLCS-YOLO model and the six considered general-purpose YOLO models in PMFP-based pipeline defect detection. The data in this figure are normalized to eliminate the effects of measurement scales and units. As shown in Figure 10, the proposed model outperformed the other models in terms of mAP@50, mAP@50:95, and precision while maintaining an acceptable parameter count and computational cost, thus achieving the best balance between accuracy and efficiency. Although YOLOv12n and YOLOv13n exhibited lower parameter counts and computational costs than the proposed model, they exhibited insufficient accuracy.

#### 3.5.2. Comparison of the Proposed Model with Representative Detection Models

To verify the comprehensive performance of DLCS-YOLO in PMFP-based pipeline defect detection, several representative detection models were selected for comparison, including improved YOLO models, D-FINE-N, and RT-DETR-L. Each model was retrained and tested on the dataset used in this study based on its official implementation and recommended configuration. For models with different architectures, parameter settings suitable for their network structures and training strategies were adopted, and performance evaluation was conducted after stable convergence was achieved.

In Table 6, P, R, F1, mAP@50, and mAP@50:95 are reported as the mean ± standard deviation of three independent training runs. The proposed DLCS-YOLO model exhibited the highest P, R, F1, mAP@50, and mAP@50:95 values among all compared models. The mAP@50:95 value of the proposed model (64.7%) was 10.6%, 4.6%, 1.8%, 2.9%, 1.0%, and 0.8% higher than those of the FT-YOLOv11, AHE-YOLO, WTAD-YOLO, YOLO11-FGA, D-FINE-N, and RT-DETR-L models, respectively. Moreover, the parameter count and computational cost of the proposed model remained within a reasonable range without substantial increases, ensuring that it achieved a favorable balance between high detection accuracy and resource efficiency.

In conclusion, the proposed DLCS-YOLO model outperformed both general-purpose YOLO models and representative detection models in PMFP-based pipeline defect detection, achieving the best balance between detection accuracy and computational efficiency.

To further analyze the detection performance of DLCS-YOLO for different defect categories, the R, AP@50, and AP@50:95 values of the baseline YOLOv11n model and DLCS-YOLO for each category were compared, and the results are presented in Table 7. R reflects the model’s ability to detect real defects in a specific category, whereas AP@50 and AP@50:95 reflect the detection accuracy of the model for that category under different IoU threshold settings.

As shown in Table 7, the detection difficulty and improvement range varied across different categories. Compared with the baseline YOLOv11n model, DLCS-YOLO exhibited relatively obvious improvements for the indentation, exhaust valve, and patch categories. Specifically, for the indentation category, R, AP@50, and AP@50:95 increased by 7.8%, 9.7%, and 10.5%, respectively. For the exhaust valve category, the corresponding improvements were 3.8%, 8.4%, and 7.7%, respectively. For the patch category, the corresponding improvements were 1.6%, 8.0%, and 8.0%, respectively. These results indicate that the proposed structural improvements have a favorable enhancement effect on categories with obvious local abnormal features, strong background interference, or high localization difficulty.

For the metal loss category, AP@50 and AP@50:95 increased by 1.6% and 1.5%, respectively, whereas R decreased slightly. Overall, this indicates improved localization accuracy while the defect detection rate remained generally stable. For the valve category, because the baseline model had already achieved high detection results, DLCS-YOLO produced a slight improvement in AP@50:95, whereas R and AP@50 decreased slightly. For the blocked-tee and tee categories, some metrics did not improve simultaneously. In particular, AP@50:95 decreased for the tee category, indicating that these categories still have room for further optimization under the current data distribution and feature representation conditions.

### 3.6. Visualization of Detection Results

**Figure 11:**
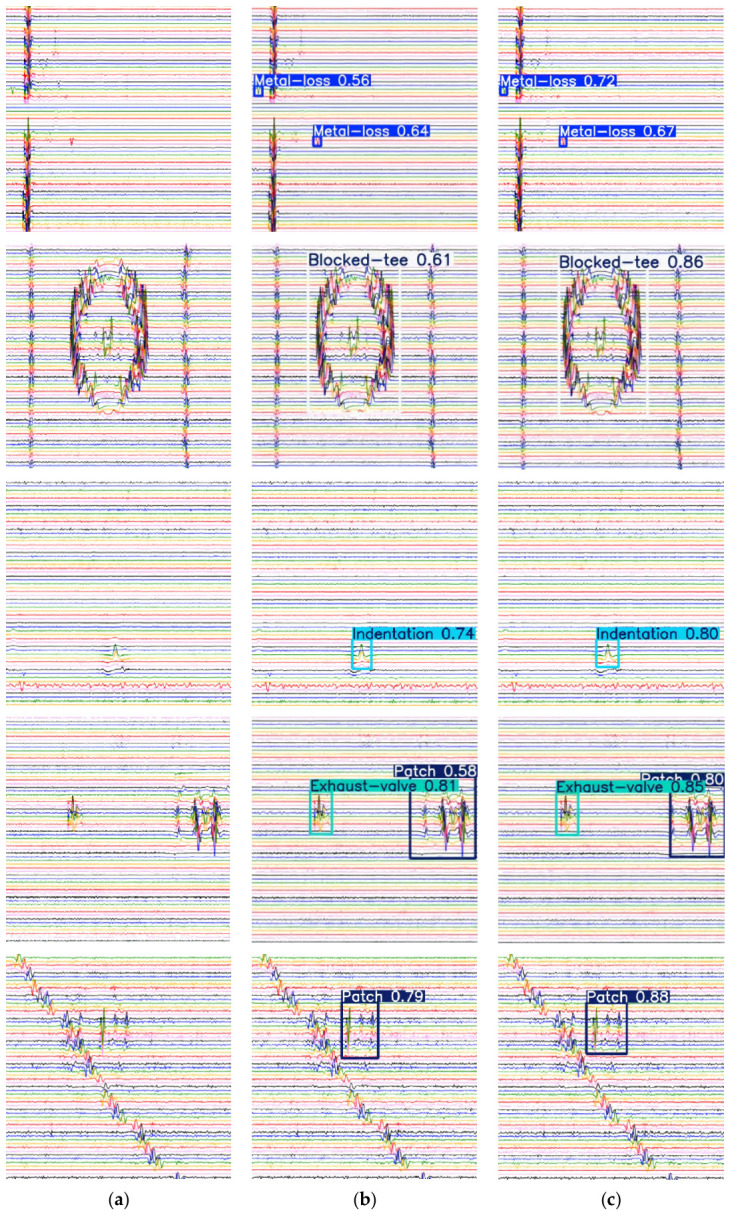
Comparison of detection results obtained with the baseline YOLOv11n model and the proposed DLCS-YOLO model. (**a**) Original image; (**b**) Detection results obtained by YOLOv11n; (**c**) Detection results obtained by our proposed DLCS-YOLO model.

To demonstrate the detection performance of the proposed DLCS-YOLO model more clearly, its detection results were compared with those of the baseline YOLOv11n model (Figure 11). The values in Figure 11 denote the confidence scores for defect recognition, with values closer to 1 indicating higher model reliability. When multiple prediction boxes were generated for the same defect in one image, only the box with the highest confidence score was retained as the final output. Compared with the baseline model, the proposed DLCS-YOLO model exhibited a higher defect identification accuracy, more precise bounding box localization, and higher confidence scores. Thus, the proposed model notably outperformed the baseline model in detection accuracy and reliability.

**Figure 12:**
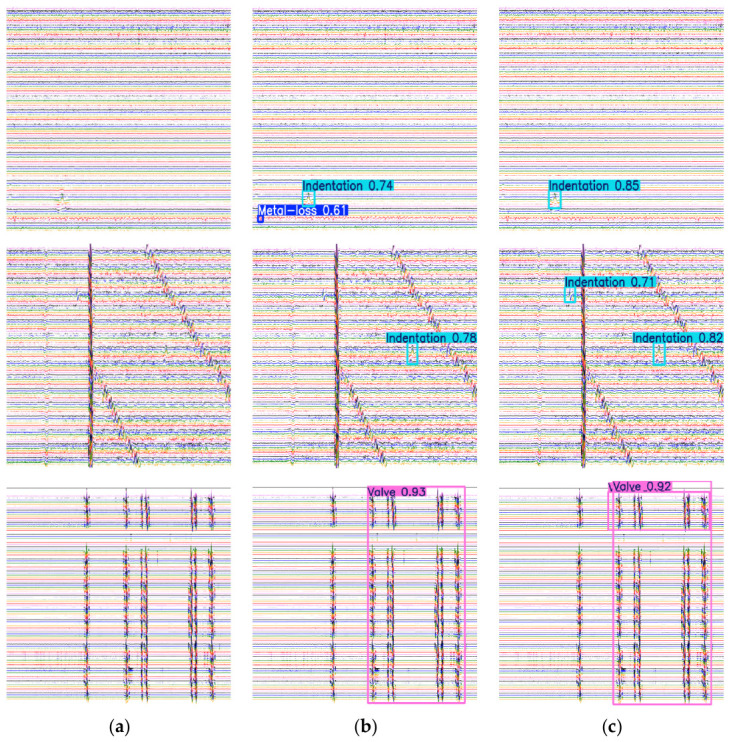
Failed detection cases of DLCS-YOLO. (**a**) Original image; (**b**) Detection results obtained by YOLOv11n; (**c**) Detection results obtained by our proposed DLCS-YOLO model.

To further objectively analyze the detection performance of DLCS-YOLO in complex PMFP images, three groups of failed detection cases were selected for visualization, as shown in Figure 12. In the first case, DLCS-YOLO correctly detected the indentation target, and the detection confidence increased from 0.74 for YOLOv11n to 0.85. However, the neighboring small-scale metal loss target was not detected. According to the labeling criteria in Table 2, metal loss usually appears as a localized downward spike or a clear peak-valley mutation within a narrow response range. Therefore, when the target scale is small, the signal response is weak, or the target appears together with a neighboring defect with a stronger response, missed detection may still occur. In the second case, the model falsely detected a single local protrusion response as an indentation. According to the labeling criteria, indentation should usually appear as several continuous upward protrusions and span more than two channel widths. A single local protrusion is therefore insufficient to form a stable indentation feature. This indicates that the model still has limitations in discriminating the continuity of local protrusions and category boundaries. In the third case, duplicate detection boxes and localization deviation occurred in the valve region. Valve targets usually have a relatively wide structural response region and are accompanied by weld-related or regular vertical fluctuation features. When the target boundary is not sufficiently clear or the structural response range is relatively wide, the model may generate repeated localization for the same target region, resulting in inaccurate bounding box localization.

These failed detection cases indicate that although DLCS-YOLO improves the overall detection performance, there remains room for further optimization in small-scale defects with weak responses, samples with similar local signal morphologies, and targets with wide or unclear boundaries. Future research will incorporate more difficult field samples and more refined feature-constraint strategies to further improve the recognition and localization ability of the model for complex PMFP images.

### 3.7. Heatmap Visualization Analysis

**Figure 13:**
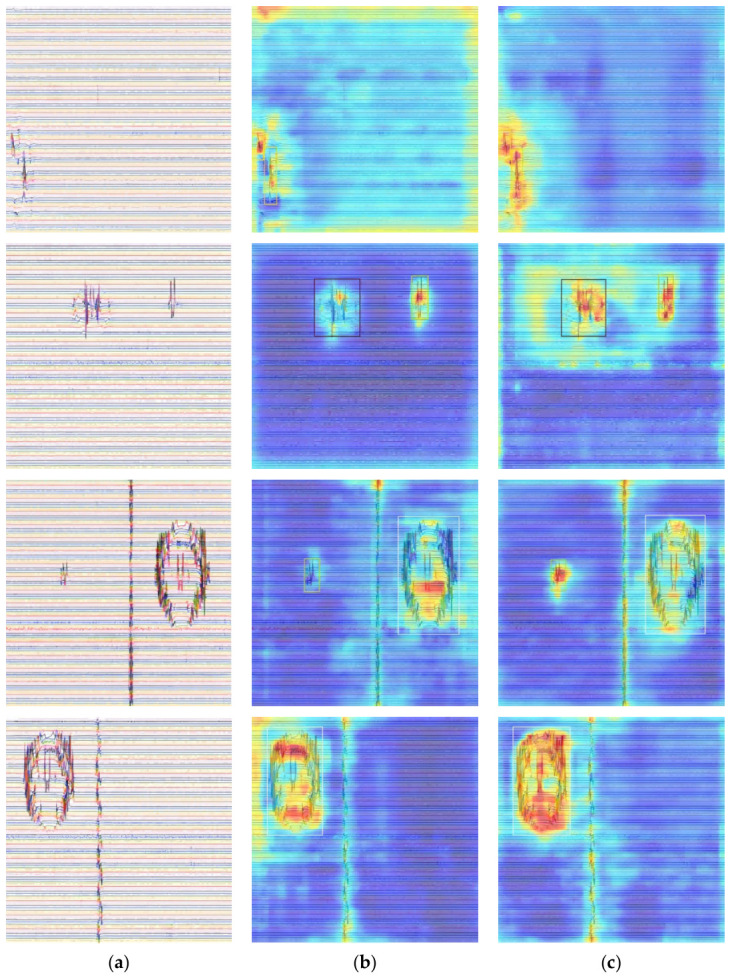
Heatmaps for the outputs produced by the baseline YOLOv11n model and the proposed DLCS-YOLO model. (**a**) Original image; (**b**) Detection results obtained by YOLOv11n; (**c**) Detection results obtained by our proposed DLCS-YOLO model. The boxes in Heatmaps indicate the target regions of interest.

To understand the advantages of the proposed DLCS-YOLO model in PMFP-based defect detection for oil and gas pipelines, its output feature maps were analyzed through heatmap visualization (Figure 13), thereby clarifying the model’s regions of interest and feature response patterns.

Figure 13a displays an input image used for defect detection, and Figure 13b,c depict heatmaps for the outputs generated by the YOLOv11n and DLCS-YOLO models under the same experimental settings. The red regions in Figure 13 indicate the key features that the models focused on. Compared with the YOLOv11n model, the proposed DLCS-YOLO model focused more accurately on pipeline defects, showing clearer feature responses in defect regions and improved performance in defect feature extraction, background suppression, and feature fusion. These results validate the practical effectiveness of DLCS-YOLO in complex PMFP-based pipeline defect detection scenarios.

**Figure 14:**
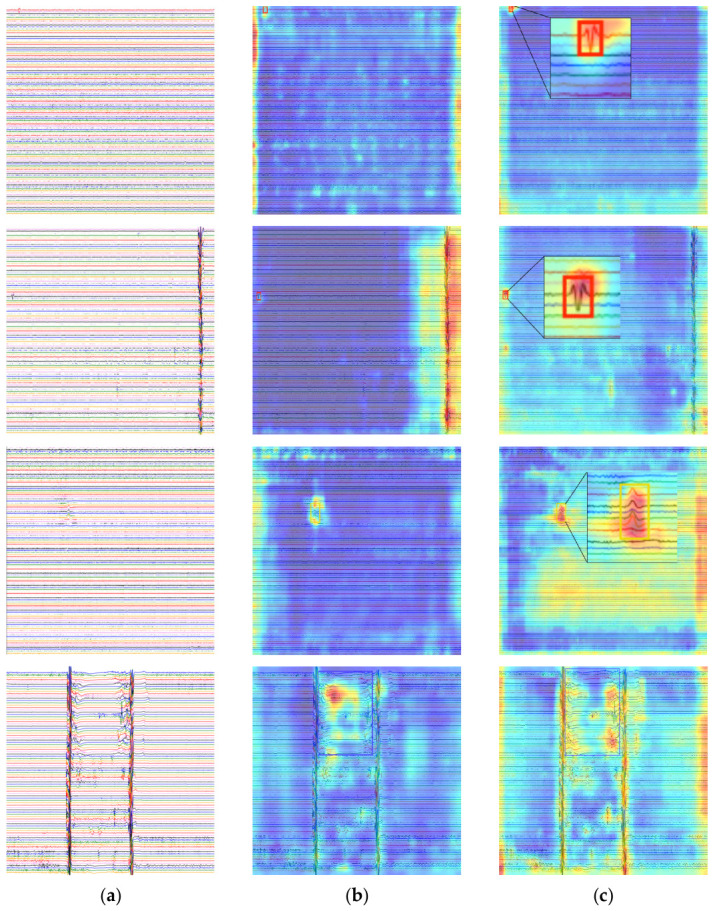
Heatmap visualization results for the SPPF-LSKA module. (**a**) Original image; (**b**) Detection results obtained by YOLOv11n; (**c**) Detection results obtained by our proposed YOLOv11n + SPPF-LSKA model. The boxes in Heatmaps indicate the target regions of interest and local enlarged regions.

To further demonstrate the effect of the SPPF-LSKA module on the focus of small-scale defects and related structural features, heatmap visualization analysis was conducted for YOLOv11n and YOLOv11n + SPPF-LSKA, and the results are shown in Figure 14.

As shown in Figure 14, the first two groups of samples are small targets belonging to the metal loss category. YOLOv11n exhibited weak attention to the metal loss regions, and the high-response regions were not sufficiently obvious. After the SPPF-LSKA module was introduced, the responses at the metal loss target locations were clearly enhanced, indicating that this module can improve the model’s ability to perceive small-scale metal loss features. The third group of samples belongs to the indentation category. YOLOv11n also showed insufficient attention to this target region, whereas YOLOv11n + SPPF-LSKA formed a more obvious high-response region at the indentation location, indicating that this module also enhances relatively small-scale indentation features. The fourth group of samples belongs to the tee category. YOLOv11n mainly focused on local responses within the target region. After the SPPF-LSKA module was introduced, the model showed a more complete attention range covering the tee target and its adjacent related response regions, with a more sufficient coverage of high-response regions. This indicates that the SPPF-LSKA module can enhance the model’s ability to perceive target-related detailed features while expanding the effective receptive field. These results are consistent with the ablation results in Table 4, further demonstrating that the SPPF-LSKA module can enhance the model’s attention to small-scale defects and target-related detailed features.

### 3.8. Generalization Analysis

To examine the generalization capability of the proposed DLCS-YOLO model, its performance and that of the comparison models were assessed on the NEU-DET dataset [30]. The NEU-DET dataset, produced by Northeastern University, comprises 1800 grayscale images, with 300 samples for each of the six defect types: crazing, inclusion, patches, pitted surface, rolled-in scale, and scratches. All experimental parameters were the same as those described in Section 3.1 to ensure the fairness and rigor of the results.

**Figure 15:**
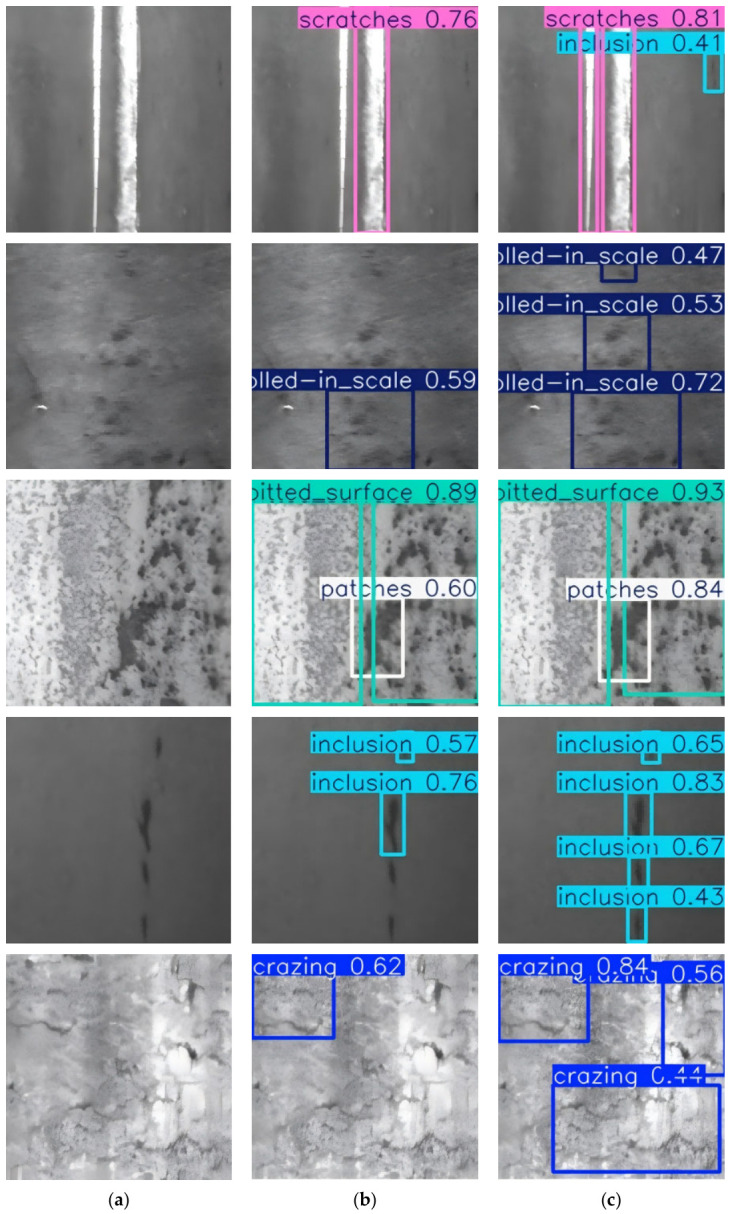
Detection results of the baseline YOLOv11n model and proposed DLCS-YOLO model on the NEU-DET dataset. (**a**) Original image; (**b**) Detection results obtained by YOLOv11n; (**c**) Detection results obtained by our proposed DLCS-YOLO model.

The experimental results presented in Table 8 and Figure 15 indicate that the precision, mAP@50, and mAP@50:95 values of the proposed model on the NEU-DET dataset were 5%, 0.7%, and 1.9% higher, respectively, than those of the baseline model. Moreover, the proposed model achieved the highest mAP@50:95 value on the NEU-DET dataset, indicating better localization accuracy under stricter IoU thresholds. These results indicate that the proposed structural improvements exhibited a certain degree of generalization capability in cross-dataset testing.

## 4. Conclusions

To address the core challenges involved in PMFP-based defect detection for long-distance oil and gas pipelines, including high false-positive rates, severe background noise interference, difficulty in identifying small-scale defects, and poor adaptability to multiscale defects, this study proposed a DLCS-YOLO model with multidimensional structural improvements by using YOLOv11n as the baseline model. In this model, the C3k2-DAttention module was introduced into the backbone network, and the original SPPF module was replaced with the SPPF-LSKA module, thereby enhancing the model’s robustness to complex background noise and its ability to capture fine-grained features of small-scale defects. Meanwhile, Context-Guided FPN was used to replace the conventional Concat feature fusion operation, effectively improving the semantic consistency and fusion efficiency of multiscale features. In addition, the SEAM was embedded into the detection head to further optimize the model’s ability to handle background interference and multiscale defects.

Experimental results indicated that DLCS-YOLO achieved mAP@50 and mAP@50:95 values of 94.5% and 64.7%, respectively, on the self-constructed dataset, which were 3.8% and 3.0% higher, respectively, than those of the baseline model. Meanwhile, the computational cost and parameter count of the model were maintained at 6.2 GFLOPs and 2.955 million, respectively, achieving a favorable balance between detection accuracy and computational efficiency. Compared with general-purpose models from YOLOv5n to YOLOv13n and several representative detection models, DLCS-YOLO exhibited certain advantages in the main detection metrics, further verifying the effectiveness of the multimodule collaborative improvement strategy.

This study provides a high-accuracy practical solution for pipeline defect detection based on PMFP data. The targeted structural improvement strategy also provides a useful reference for object detection tasks involving small targets and complex industrial scenarios, and has important engineering application value for promoting intelligent integrity management of oil and gas pipelines. In future work, the dataset scale for complex scenarios and small-scale defects will be further expanded, and deployment validation on edge-computing industrial inspection hardware will be conducted. On this basis, the model architecture will be further optimized to reduce computational redundancy, and the inference latency, runtime resource consumption, and deployment adaptability of the model in real-time online detection will be systematically evaluated, thereby improving its engineering applicability in actual pipeline inspection equipment.

## Figures and Tables

**Table 1:** Fixed experimental parameters.

Parameters	YOLO-Series	RT-DETR-L	D-FINE-N
Image size	640 × 640	640 × 640	640 × 640
Epoch	500	150	160
Batch size	32	4	8
Learning rate	0.02	0.0005	0.0008
Weight decay	0.0005	0.0001	0.0001
Momentum/β1	0.937	0.937	0.9
Optimizer	SGD	AdamW	AdamW

**Table 2:** Labeling criteria for categories.

Class	Labeling Criteria
Metal loss	The region is labeled as metal loss when the signal curve shows a localized downward spike or a clear peak-valley mutation within a narrow range. This type of response usually spans approximately 1–2 channel widths.
Indentation	The region is labeled as indentation when the signal shows several continuous upward protrusions, with an overall response direction opposite to that of metal loss. This type of response usually spans more than two channel widths.
Blocked-tee	The region is labeled as a blocked tee when the signal shows a relatively wide elliptical or curved-wave response, with a downward-curving feature near the middle of the target region.
Exhaust valve	The region is labeled as an exhaust valve when the signal shows a downward peak with upward responses on both sides. The response range is relatively wide, usually spanning approximately 10–15 channel widths.
Patch	The region is labeled as a patch when the signal shows a relatively wide circular response region with a distinct downward spike inside. The response usually spans approximately 12–17 channel widths.
Valve	The region is labeled as a valve when a large rectangular or wide-range structural response appears in the image. The central region shows a clear structural response, with weld-related or regular vertical fluctuation features. The continuous and symmetric weld region is mainly used for labeling and is confirmed according to the recorded valve mileage position.
Tee	The region is labeled as a tee according to the local structural waveform variation caused by the branch connection and the symmetric weld structures on both sides.

**Table 3:** Number of images in each category of the dataset.

Class	Original		After AutoAugment		
	Images	Boxes	Train	Val	Test
Metal loss	162	199	111	35	16
Indentation	45	49	128	40	18
Blocked-tee	16	37	92	22	12
Exhaust valve	17	21	82	20	10
Patch	20	28	95	20	14
Valve	50	50	105	30	14
Tee	38	47	140	30	18
Total	348	431	753	197	102

**Table 4:** Results of an ablation experiment.

Models	P/%	mAP@50/%	mAP@50:95/%	FLOPs/G	Params/M
Baseline	89.9	90.7	61.7	6.3	2.583
+ C3k2-DAttention	89.5	93.9	63.2	6.3	2.618
+ SPPF-LSKA	91.6	91.7	62.2	6.5	2.856
+ Context-Guided FPN	89.2	91.3	62.5	6.5	2.739
+ SEAM	91.2	90.3	62.2	5.8	2.491
+ C3k2-DAttention + SPPF-LSKA	92.6	91.4	63.1	6.7	2.889
+ C3k2-DAttention + Context-Guided FPN	91.5	92.6	61.7	6.5	2.774
+ C3k2-DAttention + SEAM	90.7	90.9	61.8	6.6	2.782
+ C3k2-DAttention + SPPF-LSKA + Context-Guided FPN	92.8	93.7	63.1	6.9	3.055
DLCS-YOLO	93.0	94.5	64.7	6.2	2.955

**Table 5:** Performance of the proposed DLCS-YOLO model and different general-purpose YOLO models in PMFP-based pipeline defect detection.

Models	P/%	R/%	F1/%	mAP@50/%	mAP@50:95/%	FLOPs/G	Params/M
YOLOv5n	92.4 ± 0.5	85.8 ± 0.3	89.0 ± 0.2	91.2 ± 0.4	60.4 ± 0.5	5.8	2.183
YOLOv8n	87.6 ± 0.5	89.9 ± 0.3	88.7 ± 0.4	90.4 ± 0.3	61.7 ± 0.1	6.8	2.685
YOLOv10n	89.5 ± 0.2	84.3 ± 0.3	86.8 ± 0.2	88.6 ± 0.2	60.6 ± 0.1	8.2	2.697
YOLOv11n	89.9 ± 0.4	88.7 ± 0.2	89.3 ± 0.3	90.7 ± 0.2	61.7 ± 0.3	6.3	2.583
YOLOv12n	87.0 ± 0.1	84.0 ± 0.2	85.5 ± 0.1	86.5 ± 0.3	58.3 ± 0.2	6.8	2.509
YOLOv13n	85.3 ± 0.2	86.6 ± 0.4	85.9 ± 0.1	87.7 ± 0.2	62.2 ± 0.3	6.2	2.449
DLCS-YOLO	93.0 ± 0.2	90.1 ± 0.3	91.5 ± 0.2	94.5 ± 0.3	64.7 ± 0.1	6.2	2.955

**Table 6:** Performance of the proposed DLCS-YOLO model and different representative detection models.

Models	P/%	R/%	F1/%	mAP@50/%	mAP@50:95/%	FLOPs/G	Params/M
FT-YOLOv11 [26]	83.4 ± 0.2	82.2 ± 0.2	82.8 ± 0.1	82.6 ± 0.3	54.1 ± 0.5	7.9	2.556
AHE-YOLO [27]	92.1 ± 0.3	87.6 ± 0.2	89.8 ± 0.3	92.3 ± 0.1	60.1 ± 0.3	4.6	3.512
WTAD-YOLO [28]	91.0 ± 0.3	88.7 ± 0.1	89.9 ± 0.1	91.6 ± 0.2	62.9 ± 0.1	6.3	2.321
YOLO11-FGA [29]	89.7 ± 0.4	88.6 ± 0.3	89.1 ± 0.4	91.5 ± 0.3	61.8 ± 0.4	8.7	3.714
D-FINE-N	92.7 ± 0.1	89.6 ± 0.4	91.1 ± 0.3	93.9 ± 0.3	63.7 ± 0.3	7.1	3.725
RT-DETR-L	92.2 ± 0.5	89.5 ± 0.5	90.9 ± 0.4	93.2 ± 0.2	63.9 ± 0.2	103.5	31.998
DLCS-YOLO	93.0 ± 0.2	90.1 ± 0.3	91.5 ± 0.2	94.5 ± 0.3	64.7 ± 0.1	6.2	2.955

**Table 7:** Per-class detection results.

Class	YOLOv11n			DLCS-YOLO		
	R/%	AP@50/%	AP@50:95/%	R/%	AP@50/%	AP@50:95/%
Metal loss	81.6	89.1	37.1	81.3	90.7	38.6
Indentation	85.0	81.3	33.3	92.8	91.0	43.8
Blocked-tee	96.4	99.5	81.0	97.1	98.7	78.5
Exhaust valve	75.0	86.9	60.6	78.8	95.3	68.3
Patch	89.3	81.1	52.3	90.9	89.1	60.3
Valve	100.0	99.5	94.9	98.1	99.2	95.2
Tee	93.3	97.7	72.9	92.0	97.2	68.3

**Table 8:** Performance of the proposed DLCS-YOLO model and different comparison models on the NEU-DET dataset.

Models	P/%	mAP@50/%	mAP@50:95/%	FLOPs/G	Params/M
YOLOv5n	77.1	77.2	44.4	5.8	2.183
YOLOv8n	72.3	77.5	44.7	6.8	2.685
YOLOv10n	72.0	76.7	44.0	8.2	2.697
YOLOv11n	66.2	76.0	44.1	6.3	2.583
YOLOv12n	70.9	75.7	44.6	6.8	2.509
YOLOv13n	65.3	73.1	41.8	6.2	2.449
FT-YOLOv11	74.3	77.9	44.1	7.9	2.556
AHE-YOLO	66.6	76.2	43.1	4.6	3.512
WTAD-YOLO	77.7	77.6	44.0	6.3	2.321
YOLO11-FGA	77.0	75.3	42.7	8.7	3.714
DLCS-YOLO	71.2	76.7	46.0	6.2	2.955

## Data Availability

Data are contained within the article.

## Abbreviations

The following abbreviations are used in this manuscript:

YOLO	You Only Look Once
DLCS-YOLO	Deformable Large-kernel Context-fused Spatial-YOLO
PMFP	permanent magnetic field perturbation
C3k2-DAttention	C3k2-Deformable Attention
SPPF-LSKA	Spatial Pyramid Pooling-Fast-Large Separable Kernel Attention
Context-Guided FPN	Context-Guided Feature Pyramid Network
SEAM	Spatially Enhanced Attention Module
ViT	Vision Transformer
CycleGAN	cycle-consistent generative adversarial network
NDT	Nondestructive testing
ILI	in-line inspection
LSKA	Large Separable Kernel Attention
CGFM	context-guided fusion submodule
